# Knowledge of postnatal mothers on essential newborn care practices at the Kenyatta National Hospital: a cross sectional study

**DOI:** 10.11604/pamj.2017.28.97.13785

**Published:** 2017-09-29

**Authors:** Lucia Amolo, Grace Irimu, Daniel Njai

**Affiliations:** 1 University of Nairobi, Nairobi, Kenya; 2 Kenya Medical Research Institute, Nairobi, Kenya

**Keywords:** Neonate, essential newborn care, neonatal mortality

## Abstract

**Introduction:**

Of the 130 million babies born yearly, nearly 4 million die in the neonatal period. Kenya Demographic Health Survey (KDHS) 2014 places neonatal mortality rate at 22 deaths per 1,000 live births, well above the Sustainable Development Goal (SDG) 3 that aims to reduce these mortalities to at least 12 deaths per 1000 live births by 2030. The aim of the study was to assess maternal knowledge on selected components of essential newborn care: breastfeeding, cord care, immunisation, eye care and thermoregulation.

**Methods:**

A hospital based cross-sectional study was conducted on 380 postnatal mothers in Kenyatta National Hospital. Interviews were conducted using structured pretested questionnaires. A score of one was given for correct response and zero for incorrect. Data were analysed using SPSS version 18.

**Results:**

Modes of thermoregulation identified included kangaroo care (7%), warm room (4%) and warm clothing (93%). Almost all mothers knew of breastfeeding on demand, exclusive breastfeeding and colostrum use. Only 17.8% of mothers identified Bacillus Calmette-Guérin (BCG) and Oral Polio Vaccine (OPV) were birth vaccines. Only 4 mothers knew no substances should be applied to the cord. In logistic regression, factors significantly associated with poor knowledge included lack of education on newborn care during pregnancy, incomplete (less than 3) or no antenatal visits with an odds ratio (OR) of 3.3 (95% confidence interval (CI), 1.5 to 7.4 ), 2.5(1.5 to 4.2), 5.1(1.3 to 19.3) and p values of 0.003, 0.001 and 0.018 respectively.

**Conclusion:**

Knowledge gaps existed regarding cord care, eye care, and immunization. Mothers had good knowledge on breastfeeding practices. Those who fail to fully attend antenatal clinics should be targeted for newborn care education.

## Introduction

Neonatal mortality remains high despite a declining proportion of under five deaths [1]. Every year, nearly 40% of all under-five deaths are among newborns [1]. Almost all (99%) of these neonatal deaths occur in developing countries with the highest rates in sub-Saharan Africa (35 deaths per 1000 live births in 2010) [2]. Neonatal health remains a challenge in Kenya. Trends in under 5 mortality between 1990 and 2014 have shown a sharp decline in infant mortality rates (77 per 1000 live births in 2004 versus 52 per 1000 live births in 2014) [3]. However, neonatal mortality rates remain high, with only a marginal decline from 33 deaths per 1000 live births in 2004 to 22 deaths per 1000 live births in 2014 [3]. The Sustainable Developmental Goals provide a new strategy to reduce neonatal mortalities. SDG 3 aims to reduce neonatal mortality to as low as 12 deaths per 1000 live births by 2030. Meeting this target implies the need for implementing achievable strategies efficiently and effectively. The World Health Organization Essential Newborn Care guidelines are evidence based measures that can be used to meet SDG 3 [4]. They encompass breastfeeding, cord care, eye care, thermoregulation, management of asphyxia, recognition of danger signs, immunization and care of the low birth weight infant. Lack of knowledge, coupled with strong cultural beliefs, influence neonatal survival once the infant is at home with the primary caregiver. Simiyu et al showed that 41% of neonatal deaths in Kenyatta National Hospital occurred in the first 24 hours of admission with majority of these deaths occurring in the first week (83.5%) of admission and noted parental delayed in bringing infants to hospital contributed to these death [5]. Newborn care practices by parents immediately after birth are important determinants of neonatal mortality. The aim of this study was to assess maternal knowledge towards essential newborn care practices.

## Methods

**Study design**: A hospital based cross-sectional study was carried out in Kenyatta national Hospital (KNH) postnatal wards between July 2013 and September 2013.

**Study population**: A total of 380 postnatal mothers were selected using consecutive sampling. Postnatal mothers of neonates born in the hospital who gave informed consent were included in the study. Exclusion criteria consisted of mothers whose neonates died or were admitted to nursery immediately after birth and those with significant congenital anomalies.

**Study procedure**: Participants were interviewed on the day of discharge using structured pretested questionnaires that captured data on socio-demographic characteristics, antenatal and perinatal history and knowledge towards essential newborn care practices.

**Data collection**: The data collection was carried out by the principal investigator or the research assistant. After recruiting the study subjects, a structured pre-tested questionnaire was used to collect data. The questionnaire was administered to the mother and their responses filled in. The questionnaire consisted of both close and open ended questions addressing the following: neonate's and parents socio-demographic data; antenatal and birth history of the neonate; mother's knowledge on the WHO essential newborn care practices. Knowledge was assessed by closed ended and open ended questions.

**Data analysis and management**: Data were collected and stored in the Microsoft access database. The collected data were then coded, verified and analysed using the statistical package for social sciences computer version 18.0 software. A scoring system was used to analyse responses to closed ended questions on knowledge: 1 = Correct response (consistent with WHO essential newborn care guidelines); 0 = Incorrect response (inconsistent with WHO essential newborn care guidelines). Any mother who did not know the answer was considered to have an incorrect response. The responses for the open ended questions were summarized and descriptive statistics carried out. During analysis for factors associated with poor maternal knowledge on newborn care, the median score was used as a cut off to distinguish between poor knowledge and satisfactory knowledge. A total of 16 questions were asked to assess knowledge on various aspects of newborn care. Those scoring below the median were considered to have poor knowledge and above or equal to the median considered satisfactory knowledge. The level of knowledge was then cross tabulated against the variables of interest. The variables which were significantly associated with poor knowledge at bivariate analysis were further analysed using multivariate analysis test (multiple logistic regression) to determine factors independently associated with poor knowledge. Associations between poor knowledge and each independent variable were examined by odds ratios (OR) and 95% Confidence Interval. Statistical testing was done using Chi square tests to compare dependent variables with explanatory variables. Data were then presented using pie charts, histograms and tables.

## Results

**Socio-demographic characteristics**: A total of 380 postnatal mothers were interviewed. The mean age of the mothers was 27.8 (± 6.4) years. Majority of the women were married (75.8%). Employed women accounted for 63.9% of those interviewed. The proportion of women who had received some basic level of education was relatively high with 45.5% having received tertiary education, 48.2% secondary education and 3.9% primary education. Christianity accounted for 93.7% of the respondents' faith with the remaining 6.3% were Muslims.

**Antenatal and perinatal history**: Of the 380 mothers interviewed, 88.2% had attended antenatal clinic. The mean gestation at first visit was 5 months (± 1.6) with an average of 3.6 visits (± 1.1). Majority of the mothers were multiparous (61.1%) while 38.9% were primiparous. The median length of postnatal hospital stay was 48 hours (24-96 hours). Table 1 shows sources of information on newborn care received by mothers.

**Table 1:** Maternal education received on essential newborn care practices

Variable	Frequency (%)	
	During pregnancy	After delivery
**Received education on newborn care?**		
Yes	307 (80.8)	103(27.1)
No	73 (19.2)	277 (72.9)
**Provider of the information+**		
Doctors	157 (51.1)	5 (4.8)
Nurses	205 (66.8)	84 (81.6)
Friends	1 (0.3)	255 (67.1)
Family	0 (0.0)	125 (32.9)
**Type of information received+**		
Breastfeeding	289 (75.2)	259 (68.2)
Cord care	64 (16.8)	15 (3.9)
Eye care	6 (1.6)	0 (0)
Thermoregulation	100 (26.3)	2 (0.5)
Danger signs in newborn	33 (8.7)	6 (1.6)
Care of low birth weight	1 (0.3)	0
Immunisation	38 (10)	25 (6.6)

+ Mothers could give more than one response

**Figure 1:**
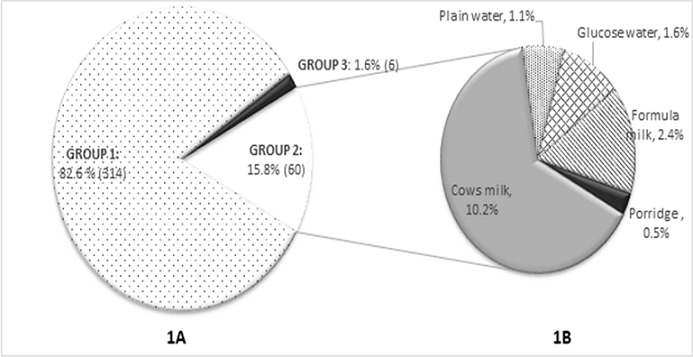
Maternal knowledge on use of prelacteal feeds in newborn: (A) shows percentage of women with knowledge on use of prelacteal feeds. Group 1 agreed that prelacteal feeds should not be given. Group 2 believed that prelacteal feeds should be given and Group 3 did not know, among Group 2; (B) shows the various prelacteal feeds identified by mothers

**Figure 2:**
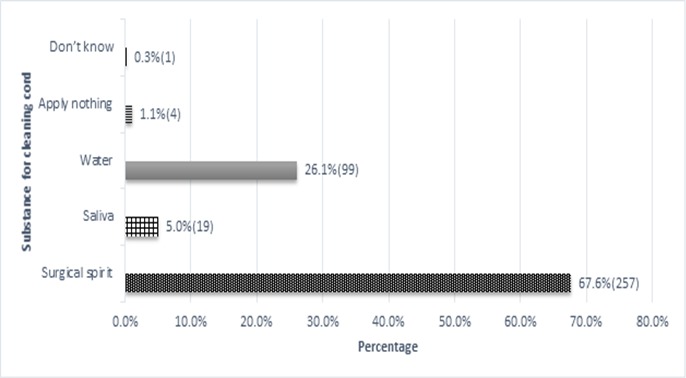
Substances identified by mothers for cleaning a soiled cord stump

**Knowledge on essential newborn care**: More than 90% of mothers knew about breastfeeding on demand, exclusive breastfeeding for 6 months and that colostrum should be given to their newborns (Table 2). The mean duration between delivery and initiation of breastfeeding reported by mothers was 15 minutes (± 5). While majority of the mothers knew that prelacteal feeds should not be given to neonates, 15.8% believed in giving prelacteal feeds (Figure 1). Almost all mothers (99.7%) were aware of the need to vaccinate neonates while 98.7% knew vaccines were given to prevent diseases. None of the mothers knew Hepatitis B vaccine was given at birth. Only 33.4% knew that BCG vaccine was for prevention of tuberculosis while 56.8% knew OPV protected the child from polio. Rooming in was identified by 94.5% of mothers as a mode of thermoregulation while 93.7% identified skin to skin contact. Only 7.1% of mothers reported they would dress the baby warmly while 4.2% knew warm room prevented heat loss. As regards cord care, 37.9% correctly stated that the stump should be uncovered. Figure 2 shows items mothers identified for cleaning a soiled umbilical cord.

**Table 2:** Frequency of maternal knowledge on selected aspects of essential newborn care

Variable	Factor level	Frequency n (%)
**Breastfeeding**		
Prelacteal feeds should be given to baby	Yes	60 (17.4)
	No	314 (82.6)
	Don’t know	6 (1.6)
Duration of exclusive breastfeeding	<6 months	7 (1.8)
	6 months	356 (93.7)
	>6 months	17 (4.5)
Babies should be breastfed on demand	Yes	377 (99.2)
	No	3 (0.8)
Colostrum should be given to baby	Yes	375 (98.6)
	No	5 (1.4)
**Immunisation**		
Vaccines prevent diseases in your baby	Yes	375 (98.7)
	No	5 (1.3)
Disease prevented by vaccine given on left forearm at birth (BCG)	Known	127 (33.4)
	Not known	253 (66.6)
Disease prevented by vaccine given orally at birth (OPV)	Known	216 (56.8)
	Not known	164 (43.2)
**Cord care**		
Umbilical stump should be left uncovered	Yes	144 (37.9)
	No	236 (62.1)
A soiled umbilical stump should be cleaned with water	Yes	99 (26.1)
	No	281 (73.9)
The cord should be left clean and dry without applying substances	Yes	4 (1)
	No	376 (99)
**Thermoregulation**		
Baby should be nursed in the same room as mother	Yes	359 (94.5)
	No	21 (5.5)
Skin to skin contact prevents heat loss in your baby	Yes	26(6.8)
	No	354 (93.2)
Warm clothing prevents heat loss in your baby	Yes	353(92.9)
	No	27 (7.1)
Warm room prevents heat loss in your baby	Yes No	16 (4.2) 364 (95.8)
**Signs of eye infection**		
Eye discharge	Yes	310 (81.6)
	No	70 (18.4)
Reddening of eyes	Yes	185 (48.7)
	No	195 (51.3)
Swollen eye	Yes	127 (33.4)
	No	253(66.6)

**Factors associated with poor maternal knowledge on newborn care**: Univariate analysis showed that primiparous had significant poorer knowledge compared to multiparous, OR 5.1 (95% CI 3.2-8.1) as were unmarried mothers, OR 2.7 (95% CI 1.7-4.4), both factors having p values less than 0.001. Poor knowledge was also associated with those who had lower than tertiary level of education; OR 6.1 (95% CI 2.5-15.1) for primary education and OR 3.3 (95% CI 2.0-5.2) for secondary education with p values both less than 0.001. Non-attendance of antenatal clinic, OR 25.8 (95% CI 9.4 to 70.6); incomplete ANC visits, OR 3.4 (95% CI 2.0-5.3); lack of education on essential newborn practices during pregnancy, OR 11 (95% CI 5.9 to 20.4) and unemployment OR 4 (95% CI 2.6-6.3) were all associated with increased chance of mothers having poor knowledge. All these factors were significant with a p value less than 0.001. In multivariate analysis to control for confounding effect, factors associated with poor knowledge included incomplete (less than 3) or no ANC visits and lack of education on newborn care during pregnancy (Table 3).

**Table 3:** Factors independently associated with poor knowledge (logistic regression)

Variable	OR (95% CI)	P-value
**Number of ANC visits**		
None	5.1 (1.3-19.3)	0.018
1-3	2.5 (1.5-4.2)	0.001
4 or more	1.0	
**Education on newborn care practices during pregnancy**		
Yes	Reference	
No	3.3 (1.5-7.4)	0.003

## Discussion

Combating neonatal morbidity and mortality requires equipping mothers with correct knowledge on newborn care to ensure appropriate practices. Table 1 showed that majority of maternal education on newborn care was provided during the antenatal period (80.8%) compared to the postnatal period (27.1%). Senarath et al showed that 75% of Sri Lankan mothers had received antenatal education, which was slightly lower than our findings [6]. A study done in Laos showed that antenatal education in expectant mothers resulted in sustained improvement in knowledge of newborn care in the postnatal period [7]. Study findings (Table 1) further revealed that the main source of information was from nurses (66.8%), followed by doctors (51.1%) during the antenatal period. In agreement with this are these are the KDHS 2014 figures which showed that 95.4 percent of women in Kenya receive antenatal care from a medical professional, either from nurses and midwives (64.3%) or doctors (31.1%) [3]. This implies that mothers in our setting rely principally on health care providers for information on newborn care rather than family and peers. A key finding in our study was that there was a significant drop in provision of information on all components of essential newborn care postnatally compared to antenatally. These discouraging results may be due an increasing trend towards a short postnatal hospital stay with inadequate time for maternal education. Majority of the education provided was related to breastfeeding with 75.2% of mothers educated antenatally and 68.2% postnatally. We noted a relatively low dissemination of information on other components of essential newborn care, with less than 10% of mothers educated on eye care, immunisation, danger signs or care of the low birth weight. Table 2 shows breastfeeding knowledge was encouraging with more than 90% of mothers aware of early and exclusive breastfeeding and breastfeeding on demand. These findings were similar to those of Sri Lankan postnatal mothers with more than 90% aware of breastfeeding on demand as well as early and exclusive breastfeeding [6]. A Ghanaian study suggesting all-cause neonatal mortality could be reduced by 16.3% if all infants initiated breastfeeding on day 1 of life and by 22.3% if initiation took place within the first hour [8]. Almost all mothers (99%) knew that colostrum should be given to their babies. These figures are relatively high compared to an Indian study where strong cultural beliefs hampered the use of colostrum with knowledge rates of 56% [9].

Our study revealed that 15.8% of mothers believed that prelacteal feeds should be used (Figure 1) which was more encouraging than a nepal study that showed 90% of mothers had no knowledge on prelacteal feeds [10]. All except two mothers agreed that breastfeeding should be conducted both day and night while 82% agreed that mixed feeding was a bad practice. Kloeblen-Tarver showed a direct correlation between maternal attitude and optimal breastfeeding practices [11]. KNH is a Baby Friendly certified hospital which could explain good knowledge levels towards breastfeeding. The cord stump is a potential source of infection if not adequately cared for. Table 2 shows 99% of mothers agreed a dirty cord could cause infection and that a previously used razor blade should not be used to cut the cord. Only 26.1% of those interviewed agreed with WHO recommendation of cleaning a soiled umbilical stump with water and only 4 mothers agreed that the umbilical cord should be left clean and dry without applying any substances. Majority of mothers (67.2%) thought that surgical spirit was appropriate for cleaning the soiled cord (Figure 2). A study done in Sri Lanka showed similar findings with majority of mothers (76.2%) also of the opinion that surgical spirit was best used to clean the soiled cord [6]. Variation in opinions among postnatal mothers is due to a lack of consensus among healthcare providers on the best practice for cord care. A Cochrane meta-analysis showed that there was no significant advantage of use of antibiotics and antiseptics over keeping the cord clean and dry in high income setting [12]. In the present study, almost all mothers were aware of the need to vaccinate their neonates. An interesting finding was none of the mothers knew of Hepatitis B vaccine while only 17.8% knew of both BCG and OPV vaccine. In Kenyatta National Hospital, nurses trained in immunisation visit the postnatal wards daily and offer both OPV and BCG vaccines to newborns. Hepatitis B vaccine is not routinely given in the hospital which could explain the lack of knowledge on this vaccine among the mothers. Findings also suggest poor dissemination of immunisation information to mothers by healthcare providers.

Table 1 shows less than 10% of mothers were educated on neonatal danger signs. Senarath also found that only 11% of Sri Lankan expectant mothers were educated on danger signs [6]. The danger signs recognised by majority of mothers were jaundice, fever, irritability, difficulty in breathing, diarrhoea and vomiting. Eye discharge was recognized by 81.6 % of mothers while less than half recognized reddening of eyes (48.7%) and swollen eyes (33.7%) as a sign of eye infection. A Bangladesh showed that an even lower proportion of 5% recognized conjunctivitis as a danger signs [13]. Almost all mothers (96.6%) recognised hyperthermia as a danger sign while only 48% recognised hypothermia. Senarath also found that hypothermia was less recognised compared to fever [6]. Studies in developing countries have shown discouraging results on knowledge of danger signs among postnatal mothers [14]. This disparity in knowledge on danger signs in our setting could translate to significant neonatal morbidities and mortalities if not addressed. Multivariate analysis showed the main predictors of poor maternal knowledge included lack of maternal education on newborn car and lack of antenatal care (Table 3). Antenatal clinic attendance was a significant predictor of poor knowledge in sharp contrast to a Sri Lanka study which showed no association between the two [6]. Findings suggest that in our setting, antenatal clinics provide an opportunity to educate mothers on newborn care.

**Study limitations**: This study was a based on reported rather than observed knowledge towards newborn care practices. There was therefore a risk that mothers may report what was expected of them but their actual practices may be different. Lack of a universal census on definition of good or poor knowledge posed a challenge in the study. As the study was carried out among postnatal mothers in Kenyatta National Hospital, findings may not be generalised to the whole country.

## Conclusion

Most maternal education on essential newborn care was received during pregnancy. Maternal education on essential newborn care was unsatisfactory with regards to eye care, care of the low birth weight, thermoregulation, immunization and danger signs in the neonate. Knowledge gaps existed among postnatal mothers with regards to eye care, cord care and immunisation. Poor knowledge on essential newborn care practices was associated with unemployment, first time mothers and those who fail to attend antenatal clinic. More focus should be placed when educating these vulnerable groups. Health education on essential newborn care practices should be integrated into routine antenatal services and re-emphasised in the postnatal period to help improve maternal knowledge and attitude towards essential newborn care practices.

### What is known about this topic

- Data on maternal knowledge and on newborn care at the point of hospital discharge is lacking in our setting;
- Timely and appropriate interventions in the first 28 days of life reduce neonatal mortalities which remain the major contributor of under five deaths;
- WHO essential newborn care guidelines are simple measures that can be used by primary caregivers in resource limited settings to ensure good neonatal outcomes.

### What this study adds

- Maternal education on essential newborn care was received primarily during pregnancy and unsatisfactory with regards to eye care, care of the low birth weight, thermoregulation, immunization and danger signs in the neonate. Knowledge gaps existed among postnatal mothers with regards to eye care, cord care and immunization;
- Poor knowledge on essential newborn care practices was associated with those who fail to attend antenatal clinic. More focus should be placed when educating these vulnerable groups;
- Health education on essential newborn care practices should be integrated into routine antenatal services and re-emphasised in the postnatal period to help improve maternal knowledge and towards essential newborn care practices.

## Competing interests

The authors declare no competing interests.
